# Association between *NFKB1* −94ins/del ATTG Promoter Polymorphism and Cancer Susceptibility: An Updated Meta-Analysis

**DOI:** 10.1155/2014/612972

**Published:** 2014-05-07

**Authors:** Xiao Yang, Pengchao Li, Jun Tao, Chao Qin, Qiang Cao, Jinbao Gu, Xiaheng Deng, Jun Wang, Xuzhong Liu, Zijie Wang, Bian Wu, Min Gu, Qiang Lu, Changjun Yin

**Affiliations:** Department of Urology, The First Affiliated Hospital of Nanjing Medical University, Nanjing 210029, China

## Abstract

Nuclear factor-**κ**B is associated with the pathogenesis of numerous malignancies, and the functional polymorphism −94ins/del ATTG (rs28362491) in the human *NFKB1* gene is associated with cancer risk. Previous studies on the association between the −94ins/del ATTG polymorphism and cancer risk reported conflicting results. To clarify this relationship, we performed a meta-analysis of 21 case-control studies involving 6127 cases and 9238 controls. We used pooled odds ratios (ORs) with their 95% confidence intervals (95% CIs) to assess the association. We found that the *NFKB1* promoter −94ins/del ATTG polymorphism was significantly associated with cancer risk in four genetic models (ins/ins versus del/del, OR = 1.47, 95% CI = 1.11–1.93; dominant model, OR = 1.26, 95% CI = 1.03–1.53; recessive model, OR = 1.26, 95% CI = 1.05–1.51; ins allele versus del allele, OR = 1.19, 95% CI = 1.05–1.35). Stratified analyses revealed a significant association between the polymorphism and ovarian, oral, and prostate cancers. Similar results were determined in an Asian population and not in a Caucasian population. Thus, our results suggested that the polymorphism can contribute to cancer risk. Moreover, the polymorphism can exert race- and cancer-specific effects on cancer risk. Further large-scale and functional studies are necessary to elucidate this possible effect.

## 1. Introduction

Cancer is a major public health problem worldwide; it is the primary and secondary causes of death in economically developed and developing countries, respectively [1]. The global concern on cancer continues to intensify as a result of the aging and expanding world population and the increasing adoption of cancer-causing habits. The mechanism of carcinogenesis remains largely unknown although genetic susceptibility is a known possible explanation for the interindividual variation in cancer risk [2].

Nuclear factor-*κ*B (NF-*κ*B) was initially identified in 1986 as a transcription factor which binds to a 10 bp DNA element in kappa immunoglobulin light-chain enhancer in B cells [3]. The NF-*κ*B family consists of p50 (NF-*κ*B1), p52 (NF-*κ*B2), p65 (RelA), c-Rel (Rel), and RelB. The major form of NF-*κ*B is a heterodimer of the p50 and p65/RelA subunits which are encoded by the* NFKB1* and* NFKB2* genes, respectively [4]. The human* NFKB1* gene is mapped to chromosome 4q24 and encodes a 50 kDa DNA-binding protein (p50) that can act as a master regulator of inflammation and cancer development [5–7].

A common insertion/deletion polymorphism (−94ins/del ATTG, rs28362491) in the promoter region of the* NFKB1* gene elicits a regulatory effect on the* NFKB1* gene [8]. A previous meta-analysis concluded that the deletion allele serves as a risk or protective allele for cancer susceptibility in Caucasian or Asian populations, respectively; however, it revealed no association between the polymorphism and cancer risk [9]. An increasing number of studies have assessed the association between the* NFKB1* promoter −94ins/del ATTG polymorphism and cancer risk [10–12]. However, these studies obtained conflicting results. Therefore, we collected all available data to perform an updated meta-analysis that generates a precise estimation to comprehensively and objectively investigate the association between the* NFKB1* promoter −94ins/del ATTG polymorphism and cancer risk.

## 2. Materials and Methods

### 2.1. Search Strategy and Identification of Relevant Studies

**Figure 1:**
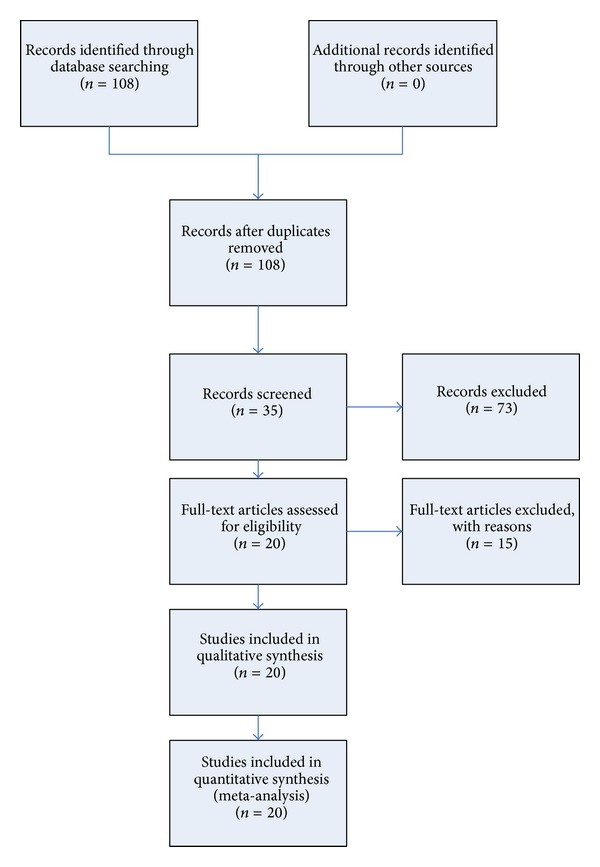
Study selection process.

A comprehensive literature search for relevant articles published (last search updated in September 15, 2013) in PubMed (http://www.ncbi.nlm.nih.gov/pubmed/) was performed with the following key words: (“genetic polymorphism,” “polymorphism,” “SNP,” “single nucleotide polymorphism,” “gene mutation,” or “genetic variant”), (“neoplasm,” “cancer,” “tumor,” “carcinoma,” or “carcinogenesis”), and (“NFKB1,” “NF-*κ*B1,” “nuclear factor kappa B1,” “NF kappa B1,” or “nuclear factor *κ*B1”). The search was limited to human studies in English. All eligible studies were retrieved. The reviews and references of eligible studies were hand-searched for additional relevant publications. The most recent or complete study was selected when more than one publications contain overlapping data. A flow diagram of the study selection process is presented in Figure 1.

### 2.2. Inclusion Criteria

Case-control studies that evaluated the association of the* NFKB1* promoter −94ins/del ATTG polymorphism with cancer risk and described in detail the genotype distributions of the polymorphism in cases and controls were included in this meta-analysis.

### 2.3. Exclusion Criteria

Studies that were not for cancer research, were only case population, and were duplication of previous publication were excluded in this meta-analysis.

### 2.4. Data Extraction

Information was carefully extracted from eligible studies independently by two investigators (Xiao Yang and Pengchao Li) according to the inclusion criteria listed above, and the result was reviewed by a third investigator (Jun Tao). The following data were collected from each study: surname of first author, year of publication, ethnicity, genotyping method, source of controls, frequencies of the genotypes in cases and controls, cancer type, and Hardy-Weinberg equilibrium (HWE) of genotype distribution among controls. Ethnicity was categorised as “Asian” or “Caucasian.” Studies that investigated more than one type of cancer were regarded as individual datasets only in subgroup analyses according to cancer type. No minimum number of patients was required for this meta-analysis. Articles that reported different ethnic groups and countries or locations were considered different study samples for each category cited above.

### 2.5. Statistical Analysis

The strength of association between the* NFKB1* promoter −94ins/del ATTG polymorphism and cancer risk was estimated through pooled odds ratio (OR) with its corresponding 95% CI. Pooled ORs were calculated for insertion allele versus deletion allele, ins/ins versus del/del, ins/del versus del/del, ins/ins + ins/del versus del/del, and ins/ins versus ins/del + del/del. Subgroup stratification analyses by ethnicity and cancer type were conducted to identify the association of the −94ins/del ATTG polymorphism with cancer susceptibility.

The between-study heterogeneity of the studies included in this meta-analysis was evaluated using the *Q* and *I* ^2^ statistic tests, where *I* ^2^ > 50% indicated heterogeneity [13]. The random-effects model was selected when *I* ^2^ was significant (>50%); otherwise, the fixed-effects model was selected. The allele frequencies of the* NFKB1* promoter −94ins/del ATTG polymorphism from the respective study were determined by allele counting. In addition, a chi-square test was used to determine whether or not the observed frequencies of genotypes conform to HWE. Pooled OR in the current meta-analysis was performed by weighting individual ORs by the inverse of their variance. The significance of the pooled OR was determined by the* Z*-test. In addition to the comparison among all subjects, we performed stratification analyses by cancer type (if one cancer type contained only one studies, it was combined into the “other cancers” group) and ethnicity. Begg's funnel plot and Egger's test were adopted to evaluate the publication bias in our meta-analysis [14, 15]. All statistical analyses were performed by STATA 10.0 software (StataCorp, College Station, TX, USA).

## 3. Results

### 3.1. Eligible Studies and Meta-Analysis Databases

A total of 21 case-control studies involving 6127 cases and 9239 controls were analysed. The characteristics of all studies are presented in Table 1. The allele and genotype frequencies of the* NFKB1* promoter −94ins/del ATTG polymorphism were extracted from all eligible studies. In total, this meta-analysis included 3 bladder cancer studies, 4 colorectal cancer studies, 2 ovarian cancer studies, 2 oral cancer studies, 2 prostate cancer studies, and 8 studies with the “other cancers.” Of the 21 studies, 14 were conducted among Asians and 7 were conducted among Caucasians. All cases were clinically pathologically confirmed.

The results of HWE test for the genotype distribution in the control population are shown in Table 1. Six of the eligible studies were not in HWE [10, 11, 18, 21, 31].

### 3.2. Quantitative Synthesis

**Figure 2:**
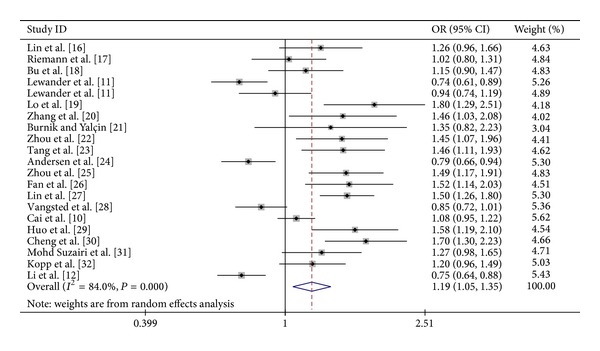
Forest plot of cancer risk associated with* NFKB1* promoter −94ins/del ATTG polymorphism (for insertion allele versus deletion allele) among all studies.

The pooled ORs of the included case-control studies revealed a statistically significant association between the* NFKB1* promoter −94ins/del ATTG polymorphism and cancer risk across the four genetic models ins/ins versus del/del, OR = 1.47, 95%, CI = 1.11—1.93; dominant model, OR = 1.26, 95%  CI = 1.03—1.53; recessive model, OR = 1.26, 95%  CI = 1.05—1.51; and ins allele versus del allele, OR = 1.19, 95%, CI = 1.05–1.35 (Table 2, Figure 2). Stratified analyses also revealed a significant association between the polymorphism and ovarian, oral, and prostate cancers in the various models. Ethnic subgroup analyses revealed significant increases in cancer risk in the four models among Asians but not among Caucasians. The results became prominent when the six studies that deviated from HWE were excluded (see Supplementary Table 1 and Supplementary Figure 1 in Supplementary Material available online at http://dx.doi.org/10.1155/2014/612972).

### 3.3. Evaluation of Publication Bias

**Figure 3:**
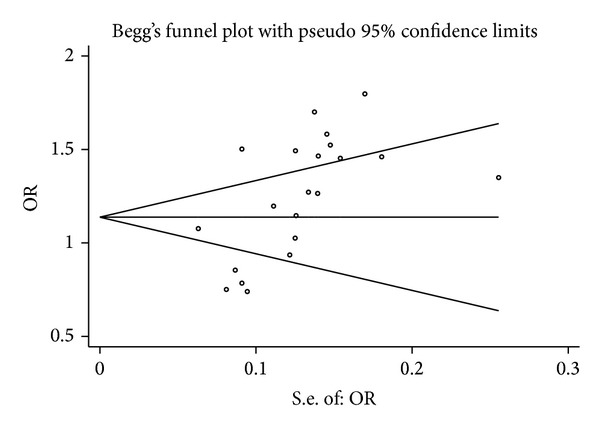
Begg's funnel plot of the association between* NFKB1* promoter −94ins/del ATTG polymorphism and cancer risk (ins allele versus del allele).

Publication bias was evaluated by Begg's funnel plot and Egger's test, and the visual asymmetry was determined in the funnel plot analysis (Figure 3). We further evaluated the publication bias in the subgroups. The results of Egger's tests for all genetic models are shown in Supplementary Table 2 (ins allele versus del allele, *P* = 0.004).

## 4. Discussion

NF-*κ*B serves important functions in pathogenetic regulation and influences cancer development and aggressiveness by enhancing tumour angiogenesis, antiapoptosis, and proliferation and by repressing immune response [7, 33, 34]. Several investigators reported the constitutive activation of NF-*κ*B in various malignancies [35, 36], including nonsmall cell lung carcinoma and colon, prostate, breast, bone, and brain cancers. p50 overexpression is frequently observed in various tumour tissues; hence, p50 is potentially involved in tumourigenesis. A polymorphism in the promoter region of* NFKB1* encoding the p50 subunit of NF-*κ*B modulates gene activity. This polymorphism has been recently reported to influence cancer risk.

A meta-analysis of all eligible studies in 2010 suggested that the deletion allele serves as a protective or risk allele for cancer susceptibility among Asians or Caucasians, respectively [9]. However, no significant association was detected for the overall population [9]. After the reported study, numerous studies further assessed the relationship between the* NFKB1* promoter −94ins/del ATTG polymorphism and cancer among Asians and Caucasians [10, 12, 32]. However, the association remains inconclusive because of the inconsistent results from the published studies. Li et al. [12] found an association between del/del genotype and bladder cancer risk but none between the polymorphism and hepatocellular carcinoma susceptibility [30].

In this study, we analysed 21 eligible case-control studies with 6127 cases and 9239 controls. The results of this meta-analysis revealed a significant association between insertion allele careers and enhanced cancer risk. The probable mechanism behind the observed association may be linked to the enhanced expression and activity of p50 (NF-*κ*B1). The insertion allele is reportedly associated with the increased promoter activity and enhanced* NFKB1* mRNA expression [8, 12, 17]. This association might influence cancer development.

The major effect of p50 (NF-*κ*B1) is mediated by its function as a component of the transcription factor NF-*κ*B, which is among the major signalling pathways involved in the cellular response to environmental stress [7]. p50 serves an important function in inhibiting cell apoptosis by modulating the expression levels of several survival genes, such as bcl-2 homologue A1 [37], PAI-2 [38], and IAP gene family [39]. Certain antiapoptosis proteins, such as Bcl-xL and Fas-associated death domain-like IL-1-converting enzyme inhibitor protein, are upregulated through the NF-*κ*B signalling pathway [40–42]. In addition, accumulated evidence illustrated that the p50 (NF-*κ*B1) signalling pathways participate in cellular proliferation by increasing IL-5 [43], promoting MAPK phosphorylation [7, 44], and modulating cyclin D1 expression [45]. Therefore, the observed association between the −94ins/del ATTG polymorphism and cancer risk can be accounted for by the insertion allele that can inhibit apoptosis and promote cellular proliferation by upregulating the expression of p50 (*NFKB1*) [8, 12, 17], which was implicated in the abovementioned mechanism.

In the stratified analyses, the increased cancer risk remained in subgroups of Asians but not in those of Caucasians. The ethnic differences in the allele frequencies may be caused by natural selection or balance to other related genetic variants. Possible differences in genetic backgrounds and gene environment may also interact with the etiology. The increased cancer risk also remained in the subgroups of ovarian, oral, and prostate cancers. This result suggested that the* NFKB1* gene might function as a prominent factor in these cancers. Therefore, further investigations are warranted to validate ethnic difference and cancer specificity in the effect of this functional polymorphism on cancer susceptibility.

This study has several limitations. First, significant between-study heterogeneity was detected in some comparisons and may be distorting the meta-analysis. Second, the genotype distribution among controls did not completely agree with HWE. However, the association between the insertion allele and cancer risk in the overall population and in the Asian population became pronounced when the six studies that deviated from HWE were excluded. Third, the studies included in the analysis used different genotyping methods with different quality control issues that may have also influenced the results. Fourth, publication bias was observed in our study, which may affect the validity of conclusion. In the stratified analysis, we found that the publication bias was significant among the Asian groups and other cancer groups but not significant among the Caucasian, bladder, and colorectal cancer groups. The sample sizes of the included studies were diverse, and most of them were insufficiently large. These conditions might partly interpret the publication bias. Finally, only three controls were population based; thus, they may not represent the general population. Therefore, the results of this study should be interpreted with caution.

In conclusion, the* NFKB1* promoter −94ins/del ATTG polymorphism is associated with cancer risk. Well-designed studies with representative sample sizes are necessary to validate these findings.

## Supplementary Material

The meta-analysis result of the studies which did not deviated from HWE.

## Figures and Tables

**Table 1:** Main characteristics of these studies included in this meta-analysis.

First Author	Year	Ethnicity	Genotyping method	SC	Genotyping cases			Controls			Cancer type	HWE
					ins/ins	ins/del	del/del	ins/ins	ins/del	del/del		
Lin [16]	2006	Asian	PCR-RFLP	HB	59	103	50	43	100	58	OSCC	0.993
Riemann [17]	2007	Caucasian	Pyrosequencing	HB	88	124	30	118	141	48	Bladder cancer	0.586
Bu [18]	2007	Caucasian	PCR-RFLP	HB	67	84	34	116	255	67	Melanoma	<0.001
Lewander [11]	2007	Caucasian	PCR-RFLP	HB	63	323	81	116	256	67	Colorectal cancer	<0.001
		Asian	PCR-RFLP	HB	50	101	42	113	266	79	Colorectal cancer	<0.001
Lo [19]	2008	Asian	PCR-RFLP	HB	62	89	31	20	62	34	Gastric cancer	0.361
Zhang [20]	2009	Asian	PCR-RFLP	HB	46	57	14	44	68	31	Prostate cancer	0.624
Burnik [21]	2009	Caucasian	PCR-RFLP	HB	18	30	2	30	58	12	GNT	0.047
Zhou [22]	2009	Asian	PCR-RFLP	HB	74	67	22	71	90	42	NC	0.177
Tang [23]	2009	Asian	PCR-RFLP	HB	89	92	26	74	108	46	Bladder cancer	0.565
Andersen [24]	2010	Caucasian	Taqman	PB	121	195	62	307	347	102	Colorectal cancer	0.801
Zhou [25]	2010	Asian	PCR-RFLP	HB	108	105	20	135	166	64	CSCC	0.297
Fan [26]	2011	Asian	PCR-RFLP	HB	78	84	17	76	103	44	Ovarian cancer	0.396
Lin [27]	2012	Asian	Taqman	HB	116	246	100	81	271	168	OSCC	0.099
Vangsted [28]	2012	Caucasian	Taqman	PB	110	163	55	665	778	253	Multiple myeloma	0.303
Cai [10]	2012	Asian	Taqman	HB	401	473	153	379	562	153	Renal cell Carcinoma	0.015
Huo [29]	2013	Asian	MassARRAY	HB	83	82	22	71	103	47	Ovarian cancer	0.399
Cheng [30]	2013	Asian	Taqman	HB	42	64	29	81	271	168	HC	0.099
Mohd Suzairi [31]	2013	Asian	PCR-RFLP	HB	35	127	75	16	138	83	Colorectal cancer	<0.001
Kopp [32]	2013	Caucasian	Taqman	PB	128	152	54	109	161	64	Prostate cancer	0.741
Li [12]	2013	Asian	Taqman	HB	189	269	151	223	324	93	Bladder cancer	0.156

GNT: Gastroenteropancreatic neuroendocrine tumors; OSCC: oral squamous cell carcinoma; CSCC: cervical squamous cell carcinoma; NC: nasopharyngeal carcinoma; HC: hepatocellular carcinoma; HB: hospital-based study; PB: population-based study; SC: source of controls; HWE: Hardy Weinberg equilibrium.

**Table 2:** Meta-analysis of the *NFKB1* −94ins/del ATTG promoter polymorphism and cancer risk.

Variables	*n* ^a^	Cases/Controls	ins/ins versus del/del		ins/del versus del/del		ins/ins + ins/del versus del/del (dominant)		ins/ins versus ins/del + del/del (recessive)		ins allele versus del allele	
			OR (95% CI)	*I* ^2^ (%)	OR (95% CI)	*I* ^2^ (%)	OR (95% CI)	*I* ^2^ (%)	OR (95% CI)	*I* ^2^ (%)	OR (95% CI)	*I* ^2^ (%)
Total	21	6127/9239	**1.47 ** **(1.11–1.93)** ^ b^	84.8	1.15 (0.97–1.37)^b^	67.7	**1.26 ** **(1.03–1.53)** ^ b^	77.5	**1.26 ** **(1.05–1.51)** ^ b^	**82.3**	**1.19 ** **(1.05–1.35)** ^ b^	84.0
Cancer types												
Bladder cancer	3	1058/1175	1.07 (0.45–2.53)^b^	90.1	1.00 (0.46–2.18)^b^	88.8	1.04 (0.46–2.33)^b^	90.7	1.04 (0.73–1.48)^b^	72.7	1.03 (0.70–1.51)^b^	89.0
Colorectal cancer	4	1275/1890	0.84 (0.47–1.50)^b^	82.9	0.93 (0.77–1.13)	0	0.88 (0.73–1.06)	0	0.89 (0.51–1.55)^b^	88.9	0.90 (0.72–1.12)^b^	76.2
Ovarian cancer	2	366/444	**2.57 ** **(1.66–3.98)**	0	**1.88 ** **(1.23–2.89)**	0	**2.17 ** **(1.45–3.25)**	0	**1.59 ** **(1.19–2.11)**	0	**1.55 ** **(1.27–1.90)**	0
Oral cancer	2	674/721	**2.10 ** **(1.54–2.87)**	33.0	**1.42 ** **(1.10–1.83)**	0	**1.59 ** **(1.25–2.03)**	3.9	**1.67 ** **(1.29–2.17)**	0	**1.43 ** **(1.23–1.66)**	6.9
Prostate cancer	2	451/477	**1.59 ** **(1.09–2.33)**	23.0	1.28 (0.89–1.84)	28.6	1.40 (1.00–1.98)	35.8	**1.33 ** **(1.01–1.74)**	0	**1.26 ** **(1.05–1.52)**	0
Other cancers	8	2303/4532	**1.72 ** **(1.13–2.61)** ^ b^	80.9	1.16 (0.88–1.53)^b^	61.3	**1.34 ** **(0.99–1.83)** ^ b^	72.4	**1.46 ** **(1.12–1.90)** ^ b^	78.0	**1.29 ** **(1.07–1.57)** ^ b^	79.9
Ethnicities												
Asian	14	4143/5169	**1.83 ** **(1.30–2.57)** ^ b^	84.8	1.23 (0.97–1.58)^b^	75.9	**1.42 ** **(1.08–1.86)** ^ b^	82.5	**1.50 ** **(1.26–1.78)** ^ b^	66.8	**1.32 ** **(1.14–1.54)** ^ b^	82.2
Caucasian	7	1984/4070	0.90 (0.64–1.27)^b^	71.2	1.00 (0.85–1.18)	18.5	0.95 (0.81–1.10)	24.0	0.90 (0.66–1.23)^b^	83.7	0.95 (0.82–1.12)^b^	70.9

^a^Number of comparisons.

^b^Random effects estimate.
